# Developmental Consequences of Early-Life Exposure and Adulticidal Effects of *Siparuna* Species Essential Oils in *Aedes aegypti*

**DOI:** 10.3390/molecules31122098

**Published:** 2026-06-15

**Authors:** Milton L. Montaño-Campaz, Javier G. Mantilla Afanador, Tarciza F. Nascimento, Joshua S. Fernandes, Mathews O. N. Novaes, Juan G. Orrego Meza, Beatriz Toro-Restrepo, Lucimar G. Dias, Patrícia F. Pinheiro, Raimundo W. S. Aguiar, Eugenio E. Oliveira

**Affiliations:** 1 Centro de Bioinformática y Biología Computacional de Colombia—BIOS, Ecoparque Los Yarumos, Manizales 170002, Caldas, Colombia; 2 Grupo de Investigación Bionat, Facultad de Ciencias Exactas y Naturales, Universidad de Caldas, Manizales 170001, Caldas, Colombia; 3 Research Institute in Microbiology and Agroindustrial Biotechnology, Universidad Católica de Manizales, Carrera 23, 60–63, Manizales 170002, Caldas, Colombia; 4 Programa de Pós-Graduação em Biotecnologia, Universidade Federal do Tocantins, Gurupi 77402-970, TO, Brazil; 5 Departamento de Entomologia, Universidade Federal de Viçosa, Viçosa 36570-900, MG, Brazil; 6 Departamento de Química, Universidade Federal de Viçosa, Viçosa 36570-900, MG, Brazil

**Keywords:** biorational insecticides, sublethal effects, fluctuating asymmetry, transient receptor potential (TRP) channels, molecular docking

## Abstract

Essential oils obtained from *Siparuna* plants, e.g., *S. guianensis* and *S. gesnerioides*, have potential for use as biorational insecticides. However, the activities of *S. gesnerioides* oils remain largely unexplored compared to *S. guianensis* oils. Using an integrative approach combining toxicological bioassays, geometric morphometrics, and in silico modeling, we assessed the adulticidal potential, selectivity, and the effects of early-life exposure to these oils on the larval susceptibility and adult wing morphometry of *Aedes aegypti*. Adulticidal assays revealed high toxicity, with *S. guianensis* (LC_50_ = 15.0 nL/mL) being 15-fold more potent than *S. gesnerioides* (LC_50_ = 233.0 nL/mL). Beyond acute lethality, early-life (i.e., eggs to L2 larvae) exposure to sublethal concentrations (*S. guianensis* = 7.4 nL/mL and *S. gesnerioides* = 118.0 nL/mL) was associated with wing morphometric disruptions and increased fluctuating asymmetry in *Ae. aegypti* adults, especially in those exposed to *S. gesnerioides* essential oil. Furthermore, early-life exposure to *S. gesnerioides* modulated L4 larvae susceptibility, which was associated with lower mortality in subsequent exposures. Selectivity assays demonstrated low acute oral toxicity in initial laboratory screenings with *Apis mellifera*, while molecular docking approaches predicted higher affinity of bicyclogermacrene and α-copaene for *Ae. aegypti* TRPV channels. Collectively, while *S. gesnerioides* oil was less acutely toxic, early-life sublethal exposures reduced fourth instar larvae (L4) susceptibility, which may have contributed to developmental instability and morphological alterations in adults. Our findings highlight the potential of *Siparuna* essential oils in mosquito management by impacting mosquito fitness beyond acute mortality.

## 1. Introduction

The global public health landscape is marked by the spread of vector-borne diseases, notably *Aedes aegypti* (Linnaeus, 1762) (Diptera: Culicidae), one of the transmitters of arboviruses such as dengue, Zika, yellow fever, and chikungunya in tropical and subtropical regions [1]. Control programs for this species have been based on actions and strategies that prevent the proliferation of breeding sites, biological control, and, predominantly, the massive application of synthetic chemical insecticides, including organophosphates, organochlorines, and pyrethroids [2,3,4]. Despite the efforts devoted to control programs, the historical dependence on synthetic insecticides has led to the emergence of population resistance [5,6]. This resistance, documented at the molecular and phenotypic levels for the main classes of insecticides, erodes the effectiveness of interventions and requires an urgent search for alternatives with novel modes of action [7,8,9,10].

In this context, the limitations of controlling *Ae. aegypti*, coupled with the development of resistance, have prompted the search for alternatives such as botanical insecticides derived from essential oils [11,12,13,14,15]. Essential oils (EOs) from aromatic plants have emerged as promising candidates due to their complex phytochemical composition, which can act on multiple molecular targets, potentially hindering the development of cross-resistance [16,17]. Their biodegradability and generally favorable safety profile for mammals and the environment position them as key components in integrated pest management strategies [18].

In the Neotropical region, the genus *Siparuna* (Aublet, 1775) (Siparunaceae) stands out for its phytochemical richness, being traditionally used in folk medicine [19], and for its insecticidal potential [12]. Previous studies have shown that the essential oils of species such as *S. guianensis* Aubl. and *S. gesnerioides* (Kunth) have high toxicity at all stages of vector development [20,21]. However, most of these studies have focused on acute and lethal toxicity, an approach that, while necessary to establish a baseline of activity, is insufficient to understand the real ecological and evolutionary impact of these bioinsecticides.

Although the acute toxicity of various plant essential oils on culicids is well-documented [22,23,24], the long-term biological consequences for surviving individuals remain frequently overlooked [25]. Investigating these impacts is crucial, as sublethal exposure can trigger physiological and morphological disruptions [26]. Such insecticide-mediated stresses (e.g., alterations in behaviors, morphology and development) can profoundly affect vector population dynamics and pathogen transmission, offering a more holistic view of a compound’s potential [27,28]. In this regard, geometric morphometric techniques can be used to detect alterations in wing venation and configuration, which serve as indicators of stress during development [29,30]. The geometric morphometry of the wing, an organ subjected to strong stabilizing pressure during development, acts as a sensitive sensor of environmental and physiological disturbances, allowing the quantification of stress induced by xenobiotics [31].

At the same time, in order to go beyond mere phenomenological description and move toward rational insecticide design, it is imperative to elucidate the underlying mechanisms of action. Furthermore, the application of in silico tools allows us to predict how the components of these oils interact molecularly [17,32]. In that regard, major components of *Siparuna* essential oils have been predicted to bind to mosquitoes’ acetylcholinesterases [21] but no other common neuronal targets have been investigated. Considering the major components of *Siparuna* essential oils have also been shown to interact with transient receptor potential (TRP) channels in other insect species [33], we used in silico approaches to investigate the potential interactions of these molecules with *Ae. aegypti* TRP channels.

Thus, this study was conducted aiming to address existing knowledge gaps through an integrative and novel approach that combines toxicological bioassays, geometric morphometrics, and in silico analysis. First, we evaluated the adulticidal potential and assessed the sublethal impacts of the essential oils of *S. guianensis* and *S. gesnerioides* on *Ae. aegypti*. The central hypothesis here is that exposure to sublethal doses of these *Siparuna* essential oils can induce developmental stress, detectable as reduced survival rates in later developmental stages and morphological disruptions (e.g., wing fluctuating asymmetry) in adults. We further assessed the selectivity potential toward the pollinator bees *Apis mellifera* and, by using in silico approaches, we predicted the potential molecular interactions among the essential oil major constituents and transient receptor potential (TRP) ion channels of the mosquitoes and honey bees.

## 2. Results

### 2.1. Yield and Chemical Composition of Essential Oils

The essential oil extraction provided distinct yields for the two species, with *S. gesnerioides* producing 0.384% and *S. guianensis* yielding 0.210%. Both essential oil types appeared as viscous, bright yellow liquids, characterized by a specific odor and a density below that of water. Chromatographic analysis revealed marked qualitative and quantitative divergence between the species (Table 1). We successfully characterized 94.62% of the *S. gesnerioides* essential oil constituents and 93.16% for *S. guianensis*, calculated from the relative peak areas. Regarding chemical classes, both essential oils are predominantly composed of sesquiterpene hydrocarbons (SH), accounting for 70.06% of the total composition in *S. gesnerioides* essential oil and 87.49% in *S. guianensis* essential oil.

### 2.2. Toxicity of Siparuna Essential Oils against Aedes aegypti Adults

**Figure 1:**
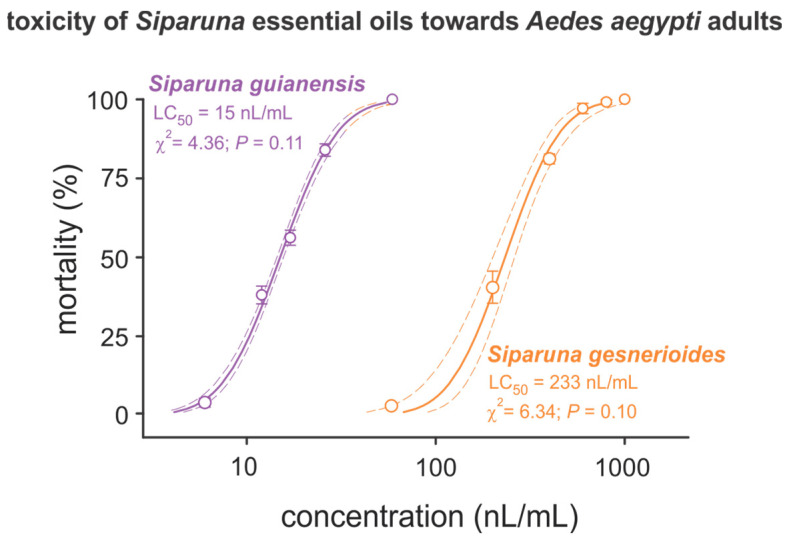
Adulticidal toxicity of dried residues of *Siparuna guianensis* and *Siparuna gesnerioides* essential oils against *Aedes aegypti*. The exposure period was 24 h. Solid lines represent mortality values estimated by the Probit model, with dotted lines indicating 95% confidence intervals. Symbols represent the mean mortality (±SEM) of four independent replicates.

Concentration–mortality results for *S. guianensis* (n = 500; χ^2^ = 4.36; *p* = 0.113) and *S. gesnerioides* (n = 600; χ^2^ = 6.34; *p* = 0.096) in adult *Ae. aegypti* after 24 h of exposure showed a satisfactory fit to the Probit model (Figure 1). The LC_50_ value for *S. guianensis* was 15.0 (14.0–16.0) nL/mL, while *S. gesnerioides* had an LC_50_ of 233.0 (201.0–261.0) nL/mL (Figure 1). There was no mortality in the control treatment; therefore, no correction using Abbott’s formula was required. Comparatively, *S. guianensis* essential oil exhibited greater toxicity against *Ae. aegypti* adults, with an LC_50_ approximately 15-fold lower than that recorded for *S. gesnerioides* essential oil.

### 2.3. Effect of Early Sublethal Exposure on Fourth-Instar Larval Susceptibility

**Figure 2:**
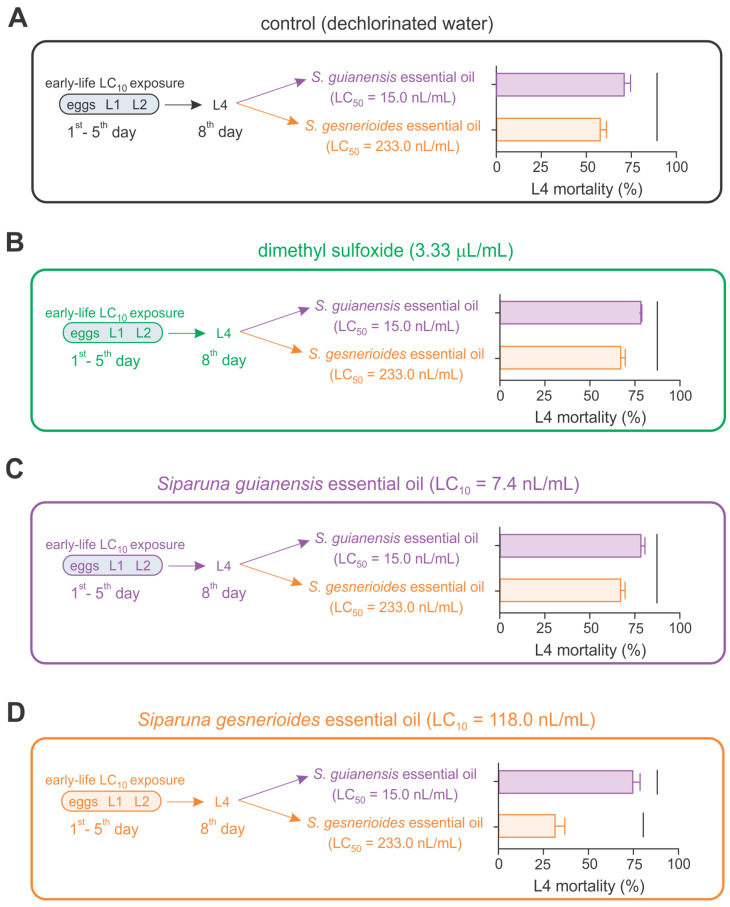
Susceptibility of *Aedes aegypti* fourth instar (L4) larvae following early-life exposure to *Siparuna* essential oils. Mortality was recorded in unexposed (**A**) larvae; larvae exposed to dimethyl sulfoxide (DMSO; 3.33 μL/mL) (**B**), and larvae exposed to the adulticidal LC_50_ (15.0 nL/mL) of *S. guianensis* (**C**) or LC_50_ (233.0 nL/mL) of *S. gesnerioides* (**D**) essential oils. LC_10_ values for early-life exposure were 7.4 nL/mL for *S. guianensis* essential oil (**C**) and 118.0 nL/mL for the essential oil of *S. gesnerioides* (**D**). Horizontal histograms represent the mean mortality (±SEM) of four independent replicates. Histograms grouped by the same horizontal lines across panels (**A**–**D**) do not differ (*p* > 0.05) by Tukey HSD post hoc comparisons.

The susceptibility of fourth-instar larvae (L4) to adulticidal LC_50_ concentrations of *Siparuna* essential oils varied significantly depending on the early-life exposure history (Figure 2). The adulticidal LC_50_ of *S. guianensis* essential oil resulted in a mortality rate of approximately 75%, regardless of previous exposure (Figure 2A–D). Notably, *S. gesnerioides* essential oil LC_50_ mediated L4 larvae mortality closer to the 50% threshold in individuals that were either previously unexposed or exposed to DMSO and *S. guianensis* essential oil (Figure 2A–C). Furthermore, a lower mortality rate was observed in L4 larvae challenged with *S. gesnerioides* essential oil when they had been previously exposed to sublethal concentrations of the same oil (Figure 2D).

### 2.4. Geometric Morphometric Analysis

The measurement error of the morphometric data was negligible, since the mean square (MS) of the error in the Procrustes ANOVA was lower than that corresponding to the Individual × Side interaction (0.0000164683 < 0.0000537868), confirming the adequate repeatability of the measurements. Procrustes ANOVA revealed highly significant effects for the Individual × Side interaction (*p* < 0.0001), both in centroid size and shape, indicating the presence of fluctuating asymmetry (FA) in the total set of samples (Control, DMSO, *Siparuna guianensis*, and *Siparuna gesnerioides*). In terms of shape, FA explained 22.93% of the total variation (SS Ind × Side = 0.06663083; SS Total = 0.29062584; *F* = 81.73; *p* < 0.0001). Additionally, the effect of the Side factor was significant, evidencing directional asymmetry, while the Individual effect confirmed the existence of significant morphological variation between specimens. Independent analysis by population showed that fluctuating asymmetry was significant in all experimental groups (Table 2). Individuals exposed to *S. guianensis* and *S. gesnerioides* essential oils showed higher percentages of variation attributable to fluctuating asymmetry compared to the control and DMSO groups. In particular, individuals that faced early development exposure to *S. gesnerioides* essential oil recorded the highest SS value (Ind × Side), suggesting differences in the relative magnitude of developmental instability between treatments.

**Figure 3:**
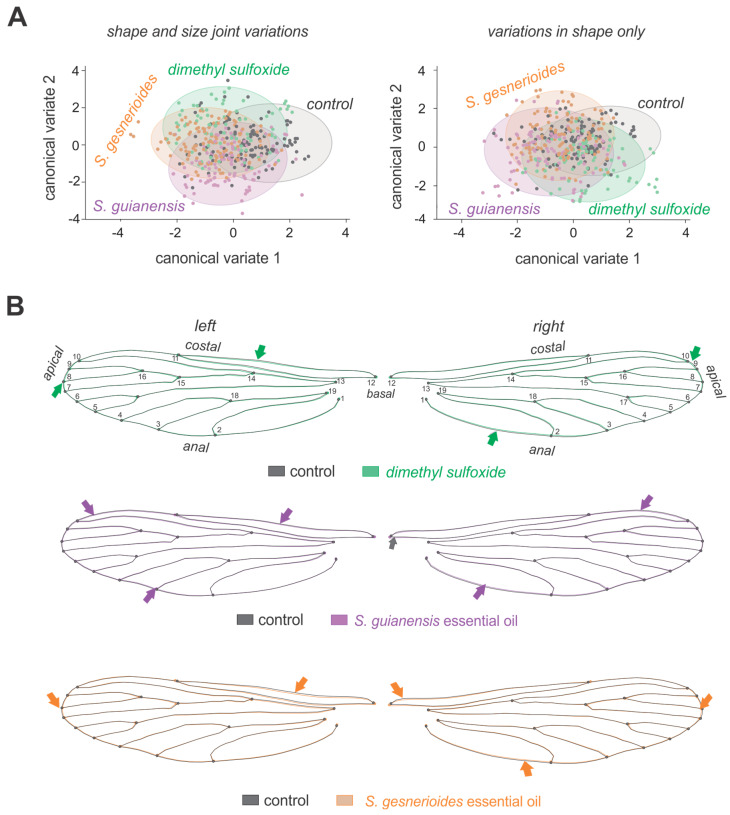
Morphometric analysis of *Aedes aegypti* wings following early-life exposure to *Siparuna guianensis* (LC_10_ = 7.4 nL/mL) and *Siparuna gesnerioides* (LC_10_ = 118.0 nL/mL) essential oils. (**A**) Canonical Variate Analysis (CVA) of wing shape and size (left) and wing shape independent of size (right), showing the discrimination of female adults surviving early exposure to the essential oils. Each point represents an individual, with ellipses indicating 95% confidence intervals for control (black), DMSO (green), *S. guianensis* (purple), and *S. gesnerioides* (orange) groups. (**B**) Visual representation of wing shape variations through wireframe diagrams. Arrows emphasize specific wing regions that underwent significant deformation.

Canonical variate analysis (Figure 3A, left side) showed significant differences in wing shape between populations (*F* = 7.4104; *p* < 0.0001). The greatest morphological divergences were observed between the Control and *S. gesnerioides*, as indicated by both Mahalanobis (2.17) and Procrustes (0.0102) distances (Table 3). Principal component analysis (PCA) showed that this variation is multivariate, with 58% of the change distributed across the first five components (PC1 = 20.2%; PC2 = 13.5%; PC3 = 9.2%). Visually, exposure to *Siparuna* essential oils, particularly *S. gesnerioides*, resulted in a contraction of the apical, costal, and anal wing regions (Figure 3B). Similar but less intense patterns of reduction were observed in the DMSO and *S. guianensis* groups. These shape differences remained consistent even after eliminating size effects. The regression of shape on centroid size was statistically significant (*p* < 0.0001), but only explained 2.28% of the total variation in shape (predicted SS = 0.00663; residual SS = 0.28399), indicating that although there is a detectable relationship between size and shape (allometry), its relative contribution is low. Consequently, most of the observed morphological variation is independent of size.

### 2.5. Selectivity of Siparuna Essential Oils for Apis mellifera

**Figure 4:**
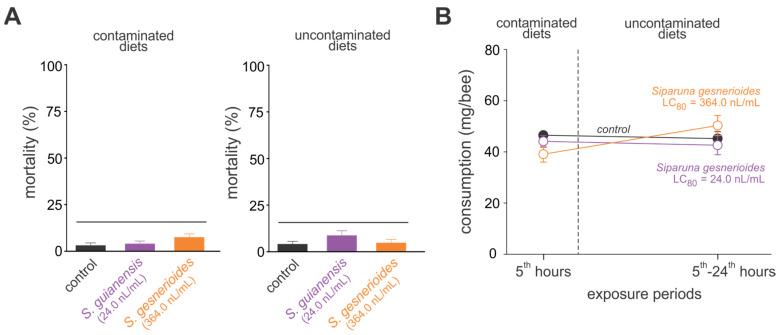
Selectivity profiles of *Siparuna* essential oils to Apis mellifera. (**A**) Mortality (%) of honey bee workers after 24 h of exposure to contaminated and uncontaminated diets. The essential oil concentrations used correspond to the LC_80_ values (24.0 nL/mL for *S. guianensis* and 364.0 nL/mL for *S. gesnerioides*) previously estimated for *Aedes aegypti*. Mortality levels in essential oil-treated groups did not differ significantly from the control. Histograms grouped by the same horizontal lines do not differ (*p* > 0.05) by Tukey HSD post hoc comparisons (**B**). Diet consumption (mg/bee) when the bees were exposed to contaminated (first five exposure hours) and uncontaminated diets (the next 19 consecutive hours). Symbols represent the average diet consumption of five biological replicates. Data are presented as mean ± standard error.

Selectivity bioassays conducted on *Ap. mellifera* using the LC_80_ concentrations of *S. guianensis* (24.0 nL/mL) and *S. gesnerioides* (364.0 nL/mL) essential oils previously determined in *Ae. aegypti* showed high insecticidal specificity for both essential oils (Table 4, Figure 4). While these concentrations caused 80% mortality in the target mosquito population, *Ap. mellifera* workers exposed to the same levels via ingestion were not significantly affected (Figure 4A). Our repeated measures analysis of variance for diet consumption showed no significant effect of time (*F* = 0.13; *p* = 0.73), and no interaction between time and the diet consumed (*F* = 1.9; *p* = 0.21) (Figure 4B).

### 2.6. Interaction of Bicyclogermacrene and Alpha Copaene Molecules and TRPV Channels of Aedes aegypti and Apis mellifera

**Figure 5:**
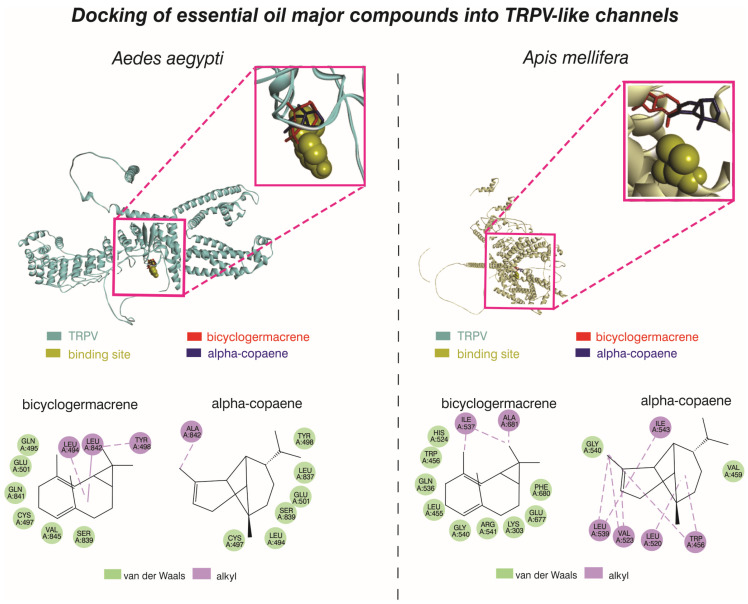
Molecular docking of *Siparuna* essential oils’ major compounds into TRPV-like ion channels of *Aedes aegypti* and *Apis mellifera*. The top panels display the 3D protein-ligand complexes for each insect species, highlighting the binding site cavities (yellow) and the spatial orientation of bicyclogermacrene (red) and α-copaene (dark blue). Insects provide a detailed view of the ligands within the receptor pockets. The bottom panels show 2D interaction diagrams for both major compounds across the two species. Intermolecular forces are categorized by color: green circles represent van der Waals interactions, while purple circles indicate alkyl or pi-alkyl bonds.

Based on our in silico computational predictions, the essential oil major constituents, bicyclogermacrene and α-copaene, can interact with TRPV channels of *Ae. aegypti* and *Ap. mellifera* through potential binding pockets. The TRPV channel from *Ae. aegypti* exhibited higher predicted binding affinity values (AutoDock Vina, version 1.5.7, kcal/mol) for bicyclogermacrene (−6.8) and α-copaene (−6.5) compared to *the Ap. mellifera* TRPV channel, which showed lower affinity values for bicyclogermacrene (−5.6) and α-copaene (−6.0) (Figure 5). The bicyclogermacrene-*Ae*. *aegypti* TRPV complex displayed predominantly hydrophobic interactions, including van der Waals contacts with GLN495, VAL845, GLN841, SER839, GLU501, and CYS497, alongside alkyl interactions with ALA842, LEU494, and TYR498. In contrast, the *Ap. mellifera* TRPV complex involved van der Waals contacts with TRP456, GLN536, LEU455, GLY540, ARG541, LYS303, GLU677, and PHE680, and alkyl interactions with ALA681 and ILE537 (Figure 5).

For α-copaene, the *Ae. aegypti* TRPV complex showed van der Waals interactions with TYR498, LEU837, GLU501, SER839, LEU494, and CYS497, and alkyl interactions with ALA842. Conversely, the *Ap. mellifera* TRPV complex exhibited van der Waals interactions with GLY540 and VAL459, alongside alkyl interactions with ILE543, LEU539, VAL523, LEU520, and TRP456 (Figure 5).

## 3. Discussion

Here, we observed that *S. guianensis* and *S. gesnerioides* essential oils possess remarkable insecticidal activity against *Ae. aegypti*. A striking differential toxicity was observed, with *S. guianensis* essential oils exhibiting significantly higher adulticidal toxicity, being approximately 15-fold more potent at the LC_50_ level than *S. gesnerioides* essential oil. Beyond acute mortality, chronic exposure to sublethal concentrations (LC_10_) was associated with complex physiological responses, reducing susceptibility in larvae pre-exposed to *S. gesnerioides* essential oil and alterations (fluctuating asymmetry) in the wing morphology of emerged adults. Furthermore, our molecular docking analyses suggest that major compounds such as bicyclogermacrene and α-copaene interact with TRPV channels, with a predictive affinity greater for the mosquito channel than for that of the honeybee *Ap. mellifera*, which correlates with the selectivity observed in bioassays.

The essential oil yields obtained for *S. gesnerioides* (0.384%) and *S. guianensis* (0.210%) essential oils are consistent with the typical extraction ranges reported for the Siparunaceae family [21,33,35,36,37]. Despite their variations in individual constituent percentages and overall yields, the fact that both *S. gesnerioides* and *S. guianensis* essential oil types exhibited predominance of sesquiterpene hydrocarbons (e.g., β-bourbonene, (E)-caryophyllene, and germacrene D) is consistent with literature reports showing that the secondary metabolism of *Siparuna* species is heavily oriented toward the synthesis of higher-molecular-weight volatile sesquiterpenes [35,37,38]. Previous studies with essential oils of *S. guianensis* and *S. gesnerioides* had already reported their larvicidal activity [12,21,39]. Here, however, we quantified the adulticidal toxicities, for the first time, of *Siparuna* species essential oils revealing a striking difference in potency that highlights the importance of intrageneric chemodiversity. Additionally, the choice of using adult-derived lethal concentrations for the larval stage bioassays was a deliberate experimental strategy to ensure consistency by using the same essential oil batch across all life stages, although we recognize that these concentrations do not represent biological equivalence between larvae and adults.

The differential toxicity observed between the essential oil of the two *Siparuna* species (LC_50_ of 15.0 nL/mL for *S. guianensis vs* 233.0 nL/mL for *S. gesnerioides*) may be related to their qualitatively and quantitatively distinct phytochemical profiles. While *S. guianensis* essential oil has a more simplified composition dominated by bicyclogermacrene (42.9%) and germacrene D (25.1%), *S. gesnerioides* essential oils exhibit a complex mixture of more than 30 compounds, including germacrene D (19.9%). Although synergistic actions among essential oil components are frequently reported [40], the greater potency of *S. guianensis* oil could potentially arise from interactions between its major constituents or the high concentration of specific active compounds, such as bicyclogermacrene. However, these specific synergistic effects remain to be experimentally confirmed through bioassays with isolated compounds. It is also worth noting that major compounds are not always responsible for the insecticidal or repellent activities of plant-based essential oils. For instance, (E)-β-farnesene, a minor component of natural pyrethrins, is of great relevance to the repellent actions of pyrethrum extracts on *Ae. aegypti* [41]. Given the presence of unidentified compounds (approximately 6.8% in *S. guianensis* essential oil and 5.4% in the *S. gesnerioides* essential oil) and considering that our identification relies on library matching and retention indices, our findings do not rule out their potential contribution to the adulticidal and sublethal effects observed in this study.

A particularly novel finding in our study is the differential response in larval susceptibility following early-life exposure. While larvae challenged with *S. guianensis* essential oil at their early developmental phases (i.e., from eggs to L2 larval instar) showed no significant change in susceptibility, those previously exposed to sublethal concentrations of *S. gesnerioides* essential oil exhibited significantly lower susceptibility at the L4 stage. Although further investigations are still required to disentangle the reasons for such physiological changes, it is hypothesized that the reduced susceptibility observed after exposure to *S. gesnerioides* essential oil may involve the activation of metabolic detoxification mechanisms, such as cytochrome P450 complex enzymes [42,43,44], glutathione S-transferases [45,46], or esterases [44,47,48]. These pathways are known to be inducible in insects after exposure to complex xenobiotics [49], and their role in the current study remains a subject for future biochemical and transcriptional validation.

The reduced susceptibility of L4 larvae following early-life exposure to *S. gesnerioides* essential oil appears to come at a physiological cost. This survival mechanism likely triggers a trade-off, which can be related to the wing deformations observed in emerged adults. This phenomenon occurs when energy resources are diverted from developmental stability to sustain costly detoxification mechanisms [50,51]. Despite the fact that larvae early-life exposed to DMSO alone resulted in adults with significant fluctuating asymmetry (approximately 21%), suggesting that the surfactant(solvent) itself may contribute to developmental stress, both essential oil treatments exhibited notably higher percentages of fluctuating asymmetry (approximately 25%). While the difference between the solvent control and oil treatments is narrow, it suggests a possible additive effect of the essential oils on developmental instability, beyond the baseline stress caused by the surfactant/solvent. Additionally, the substantial effect of DMSO alone represents a limitation in isolating the oil-specific developmental impact, requiring caution in this interpretation. Since the alterations were identified in functional costal and apical wing regions, these sublethal effects could translate into reduced flight capacity and host-seeking efficiency, directly impacting the biological fitness and vectorial capacity of the surviving population [52,53]. Fluctuating asymmetry is considered a sensitive biomarker of environmental and genetic stress, as it reflects an organism’s inability to maintain perfectly symmetrical development under disruptive conditions [53]. Although the current investigation has not addressed the mechanistic reasons in detail, the increase in fluctuating asymmetry in the wings of mosquitoes exposed to *Siparuna* essential oils indicates that these compounds, even at concentrations that do not cause mortality, may interfere with cellular and developmental processes, potentially due to their predicted neurotoxic action and the hypothesized energy cost. The alterations in the apical, costal, and basal regions of the wing (identified by ACP) could translate into functional consequences, affecting flight capacity, host seeking, and mating, which would directly impact the biological fitness of the vector [52,54,55]. While these functional impacts are hypothesized based on the literature on wing morphometrics, they remain to be empirically tested in mosquitoes surviving *Siparuna* essential oil exposures.

The hypothesis of a neurotoxic mode of action is supported by in silico studies. Our molecular docking predicted that bicyclogermacrene and α-copaene may interact with TRPV channels. The TRPV channel of *Ae. aegypti* exhibited higher predicted binding affinity values (bicyclogermacrene: −6.8 kcal/mol; α-copaene: −6.5 kcal/mol) compared to the channel of *Ap. mellifera* (−5.6 and −6.0 kcal/mol, respectively). The interactions were predominantly hydrophobic (van der Waals and alkyl) in both cases. Transient receptor potential vanilloid (TRPV) channels are known targets for certain insecticides and botanical compounds, and their modulation can cause hyperexcitability, paralysis, and death [36,56,57]. The higher affinity predicted by the mosquito channel suggests a molecular basis for the selectivity observed in bioassays with *Ap. mellifera*. Differences in the amino acid sequence of the TRPV channel binding pocket between the two species, although not explored in detail here, are the most likely cause of the differences in predicted binding energy. Our findings do not rule out that selectivity may also arise from differential susceptibility in other physiological targets, such as nicotinic acetylcholine (nAChRs), octopamine, or γ-aminobutyric acid (GABARs) receptors. Further electrophysiological assays of the essential oil major compounds and the TRPV channels are still required before firm conclusions are considered.

Selectivity tests showed that LC_80_ concentrations for the mosquito caused less than 20% mortality in honeybees, which was expected, as a previous study found *S. guianensis* essential oil to be selective against honey bees [58]. This selectivity is a crucial attribute for any new control agent, as it minimizes the impact on pollinators and other beneficial organisms [17], facilitating its integration into IPM strategies that seek a balance between pest control and biodiversity conservation [59]. Here, even when we consider the fact that the current investigation addressed a worst-scenario case by confining forager bees to small containers and allowing feeding only from a single source containing essential oil concentrations at levels of exposure significantly higher than what is typically encountered in a natural, open-field environment, our selectivity data points toward a promising preliminary safety profile compared to the target organisms (i.e., mosquito adults).

Despite the robustness of the results, certain limitations must be acknowledged. Molecular docking studies, while informative, are predictive and static. They do not consider the dynamics of protein-ligand interaction in a membrane environment, nor possible metabolisms of the compound before reaching its target [60]. Future research should employ molecular dynamics simulations to validate the stability of these complexes and perform electrophysiological assays to functionally confirm the modulation of TRPV channels by these compounds. The potential contribution of unidentified compounds to the observed effects (adulticidal toxicity and sublethal activities) remains uncertain and warrants further investigation. Similarly, toxicity tests on bees, although encouraging, considering that these bioassays were conducted under laboratory conditions with acute oral exposure, in the worst-case scenario, as they would not have the chance of evading the contaminated food source, field studies and assessments of chronic and sublethal effects (e.g., on foraging behavior) in *Ap. mellifera* and other native pollinators are needed to fully confirm their ecological selectivity.

## 4. Materials and Methods

### 4.1. Collection of Plant Material and Extraction of Essential Oil

The leaves of *Siparuna guianensis* and *Siparuna gesnerioides* were collected in the municipality of Norcasia (5°34′27″ N, 74°53′20″ W; 700 m above sea level), department of Caldas, Colombia. Taxonomic identification was confirmed by specialists at the Herbarium of the University of Caldas (Manizales, Colombia), where the reference specimens were deposited under the numbers JAO 957 (*S. guianensis*) and JASG 1522 (*S. gesnerioides*). For each species, young and mature leaves were collected from different individuals and positions within the plant to ensure the representativeness and randomness of the sample. The plant material was placed in properly labeled paper bags and transported to the Zoology Laboratory of the University of Caldas (Manizales, Colombia). The leaves were dried in the shade at room temperature and then stored in plastic bags until extraction. The essential oils were obtained at the Kupay Laboratory (Bogotá, Cundinamarca, Colombia) by steam distillation, using a 10 Kg distillation unit similar to those used on a commercial scale. Approximately 1600 g of dried leaves were used in each extraction, with a distillation time of four hours.

### 4.2. Chemical Composition of Essential Oils

For the analysis of the chemical composition of the essential oil, a solution containing 10 mg of essential oil in 1 mL of ethanol was prepared. An aliquot of 1 µL of this solution was injected into a gas chromatograph coupled to a mass spectrometer (GC–MS), model GCMS-QP2010C Ultra Mass Spectrometer (Shimadzu Corporation, Kyoto, Japan). The stationary phase used was an SPB-5 fused silica capillary column (30 m length, 0.25 mm internal diameter, and 0.25 µm film thickness), and helium was used as the carrier gas. The injector temperature was set at 220 °C and the detector at 300 °C. The initial column temperature was 40 °C, programmed to increase at a rate of 5 °C per minute until reaching a maximum temperature of 300 °C. The column flow rate was 1.60 mL/min. For compound identification, the obtained mass spectra were compared with those available in the equipment database (NIST spectral library), as well as with literature data and their respective retention indices (RI).

The retention indices (IR) were calculated according to Equation (1) for each compound and compared with literature values [34]:

(1) $$IR\;\left(X\right)=100Pz+100[\frac{\left(RTx-RTPz\right)}{\left(RTPz+1-RTPz\right)}]$$

where *X* = compound of interest; *Pz* = number of carbon atoms of the n-alkane eluting immediately before compound *X*; *RTx* = retention time of compound *X*; *RTPz* = retention time of the preceding n-alkane; and *RTPz* + 1 *=* retention time of the n-alkane eluting immediately after compound *X*.

### 4.3. Breeding Conditions for Aedes aegypti and Apis mellifera

Adult *Ae. aegypti* from the PPCampos insecticide-susceptible strain were used, maintained for eight years under controlled conditions and in an environment free from exposure to insecticides. The larvae were reared in dechlorinated water and fed daily with commercial turtle food under controlled environmental conditions (25 ± 2 °C; 60 ± 2% relative humidity; photoperiod 12:12 h light:dark) until adult emergence. Adulticide bioassays were performed with individuals 5 to 10 days old post-emergence, following the methodology previously described elsewhere [2,61]. The *Ap. mellifera* colonies used in the trials were obtained from the experimental apiary of the Federal University of Viçosa (UFV), where they are kept under health monitoring and standardized technical management. Clinically healthy colonies with young queens and active egg laying were selected, from which homogeneous experimental nuclei were established (3–5 frames with capped brood and food reserves). These microcolonies were kept in small-volume Langstroth hives under natural environmental conditions, supplemented with sucrose syrup (50%, *w*/*v*) and protein substitute when necessary. Prior to exposure to essential oils, the colonies were stabilized for at least two weeks to minimize handling stress. The treatments were applied under controlled conditions, avoiding drift between hives and cross-contamination, in order to ensure reliability in the evaluation of lethal effects.

### 4.4. Adulticidal Bioassays Against Aedes aegypti

The adulticidal activity of the essential oils of *Siparuna guianensis* and *Siparuna gesnerioides* against *Aedes aegypti* was evaluated using the methodology described by [2,6], with slight modifications. The essential oils were diluted in acetone to obtain experimental concentrations. The tested concentration ranges were 6.0–34.0 nL/mL for *S. guianensis* and 80.0–1000 nL/mL for *S. gesnerioides* essential oils. Two milliliters of each solution were dispensed into 250 mL glass bottles (internal area: 179.1 cm^2^) and rotated manually to ensure homogeneous distribution on the internal surface. The solvent was allowed to evaporate completely at room temperature, enabling uniform impregnation of the essential oil in the bottle walls. Then, 25 adult female *Ae. aegypti* (4–7 days post-emergence) were introduced. The mosquitoes were fed a 10% sucrose solution and fasted for 24 h prior to the bioassay. Mortality was recorded after 24 h of exposure. Each concentration was evaluated in four independent biological replicates, totaling 100 mosquitoes per concentration.

### 4.5. Effect of Early Sublethal Exposure on Fourth-Instar Larval Susceptibility in Aedes aegypti

*Aedes aegypti* larvae obtained from newly hatched eggs were used to evaluate the effect of early sublethal exposure on susceptibility in the fourth larval stage. The sublethal concentrations (LC_10_) for early-life exposure assays were specifically selected based on the adulticidal potency of the current essential oil batch. This approach was adopted to ensure absolute chemical consistency across all experimental stages, as essential oil compositions are known to vary significantly between different harvest batches [18]. Although larvicidal values for these species were previously reported [21], those referred to a distinct chemical profile. Therefore, using the current batch’s LC_10_ and LC_50_ referred values allowed for a strictly controlled assessment of how the same chemical matrix triggers distinct responses from larval development to adult morphology. Based on these analyses, 7.4 nL/mL was selected for *S. guianensis* essential oil and 118.0 nL/mL for *S. gesnerioides*. The organisms were exposed to these concentrations from the embryonic stage for five consecutive days. Additionally, a control treatment with dimethyl sulfoxide (DMSO; 3.33 μL/mL) and a negative control with uncontaminated dechlorinated water were included. The inclusion of DMSO control was fundamental to our experimental design as the hydrophobic nature of *Siparuna* essential oils requires a solvent carrier to ensure chemical homogeneity, stability, and bioavailability of the constituents to the larvae. This control allows for discrimination against any potential basal stress induced by the solvent from the specific toxicological effects of the essential oils. After the five-day exposure period, the treated water was replaced with contaminant-free dechlorinated water, and the larvae continued their development under standard conditions until they reached the fourth stage (L4). At this point, individuals from each previous treatment (i.e., control, DMSO, *S. guianensis* essential oil, and *S. gesnerioides* essential oil) were subjected to a susceptibility bioassay by exposure for 24 h to the LC_50_ concentrations previously determined for each essential oil (15.0 nL/mL for *S. guianensis* and 233.0 nL/mL for *S. gesnerioides*) in adult mosquitoes. Groups of 25 L4 larvae were formed using a Pasteur pipette and distributed into 250 mL glass vials containing 50 mL of the corresponding solution, each vial constituting an experimental unit. Specifically, each treatment consisted of four independent biological replicates, with 25 L4 larvae per replicate (totaling 100 larvae per group). Mortality was recorded 24 h after exposure, considering those larvae that did not move or respond to mechanical stimulation with a Pasteur pipette to be dead.

### 4.6. Analysis of Wing Fluttering Asymmetry in Aedes aegypti Exposed to Sublethal Concentrations of Siparuna Essential Oils

To evaluate the asymmetric differences induced by chronic sublethal exposures, we used *Ae. aegypti* individuals from four experimental sublethal exposure types: Control (dechlorinated water), DMSO (3.33 μL/mL), *S. guianensis* essential oil (7.4 nL/mL), and *S. gesnerioides* essential oil (118.0 nL/mL). Such sublethal concentrations (LC_10_) were previously determined from dose–response curves constructed in adults for each essential oil. The organisms were exposed to these concentrations from the embryonic stage for seven consecutive days. Subsequently, the treated water was replaced with dechlorinated water free of contaminants, and the larvae continued their development under standard conditions until the emergence of adults. In order to quantify possible alterations in wing shape associated with early exposure, fluctuating asymmetry analyses were performed using the right and left wings of adult females. The wings were carefully removed, mounted between slides and coverslips in a standardized position, and photographed with a Leica M205 C stereomicroscope equipped with a digital camera. The images were stored in JPEG format, and 19 anatomically homologous reference points per wing were digitized using tpsDIG2 v2.17 software. The criteria of positional homology, relative consistency, adequate shape coverage, and repeatability were considered during digitization.

The Cartesian coordinates (X–Y) were subjected to generalized Procrustes adjustment to extract information independent of size, position, and orientation. Measurement error was evaluated following the protocol of [51] by redigitizing a subset of samples. Fluctuating asymmetry was analyzed using Procrustes ANOVA, considering the individual effect, side effect, and individual × side interaction. The quadratic means of the interaction (MS individual × side) were used as estimators of fluctuating asymmetry. Size asymmetry was calculated as the absolute difference between the centroid sizes of the right and left wings divided by the mean centroid size. Shape asymmetry was estimated as the Procrustes distance between both wings of each individual. The data were subjected to MANOVA analysis to evaluate differences between treatments. The main patterns of morphological variation were explored using principal component analysis (PCA) based on the covariance matrix of the symmetric component of shape. Canonical variable analysis (CVA) was also performed to discriminate between treatments, and Procrustes distances between groups were calculated. Finally, the relationship between shape and centroid size (allometry) was evaluated using multivariate regression with 10,000 permutations, including 90% confidence ellipses for each group. All statistical analyses were performed using MorphoJ 2.0 software. Each treatment contained the wings (left and right) of 30 individuals that were used as independent replicates.

### 4.7. Selectivity Bioassays in Apis mellifera Foragers

We conducted oral toxicity bioassays with *Ap. mellifera* foragers to evaluate the acute oral safety of the essential oils by following procedures previously described elsewhere [62]. Briefly, we used the essential oil at concentrations equivalent to the LC_80_ for adult mosquitoes (364.0 nL/mL for *S. gesnerioides* essential oil and 24.0 nL/mL for *S. guianensis* essential oil). These essential oils were diluted in a 10% (*m*/*v*) sucrose solution containing DMSO (3.33 μL/mL). Foragers were collected from five different colonies to account for intercolonial genetic variation. To minimize colony effects, bees from each colony were equally distributed across all experimental groups. Bees were acclimatized in a BOD chamber at 32 °C and fasted for one hour prior to the assays to standardize food intake. The bioassays followed a completely randomized design with five replicates per treatment. Each replicate consisted of a 250 mL plastic container housing 10 bees (n = 50 per group). Treatments were offered in 2 mL microtubes, including a solvent control (10% sucrose + 3.33 mL/mL DMSO) and a negative control (10% sucrose only). Mortality was assessed at 5th and 24th h post-exposure, defined by the lack of response to gentle mechanical stimulation with a fine-bristled brush. This setup represents a conservative ‘forced-exposure’ scenario to rigorously test for acute oral toxicity.

### 4.8. Comparative In Silico Analysis of TRPV Channels in Aedes aegypti and Apis mellifera

Molecular docking analyses were performed to evaluate the interactions between the major sesquiterpene compounds, bicyclogermacrene and α-copaene, the major compounds of the essential oils of *S. guianensis* and *S. gesnerioides*, and the transient receptor potential vanilloid (TRPV) channels of *Ae. aegypti* and *Ap. mellifera*. The amino acid sequences of (TRPV) receptors from *Ae. aegypti* and *Ap. mellifera* were retrieved from the National Center for Biotechnology Information (NCBI) database under accession numbers QSH48591 (*Ae. aegypti*) and XP_026296684 (*Ap. mellifera*). The three-dimensional structure of the TRPV channel of *Ae. aegypti* was generated using the SWISS-MODEL server. During the modeling process, the platform automatically selected as a template a structure predicted by AlphaFold, corresponding to *Anopheles culicifacies* (accession: A0A182MF93). The resulting model showed the Ramachandran plot [63,64] analysis indicated that 94% of residues were located in favored regions, supporting good stereochemical quality, a Global Model Quality Estimation (GMQE) score of 0.76 [65]. For *Ap. mellifera*, the TRPV structure (accession: A0A7M7FZ19) was predicted by an AlphaFold corresponding to the insect structure. Structural validation showed a GMQE value of 0.73, with 90% of residues located in favored regions of the Ramachandran plot, indicating acceptable structural quality. Protein structures were energy-minimized using YASARA to improve stereochemical quality and reduce potential steric clashes [66]. Subsequently, proteins were prepared for docking using AutoDock Tools [67]. During this process, water molecules were removed, polar hydrogens were added, Gasteiger charges were assigned, and the prepared protein structures were then converted into PDBQT format. After that, ligand structures for bicyclogermacrene and α-copaene were obtained from the PubChem database in SDF format.

These ligand structures were converted to PDB format using PyMOL, version 2.0 [68] and prepared for docking using AutoDock Tools [67,69]. During this process, polar hydrogens were added, Gasteiger charges were assigned, and the ligands were converted into PDBQT format. Molecular docking simulations were carried out using AutoDock Vina [70]. The docking search space was defined to cover the transmembrane region of the TRPV channel. For *Ae. aegypti*, the grid box was centered at coordinates (center_x = 1.378, center_y = 1.286, center_z = −1.824) with dimensions of 40 × 40 × 40 Å. For *Ap. mellifera*, the grid box was centered at coordinates (center_x = −3.436, center_y = 0.517, center_z = 3.230) with dimensions of 40 × 40 × 40 Å. Docking simulations were performed using an exhaustiveness value of 8, generating up to 9 binding poses per ligand. The energy range was set to 4 kcal/mol. The conformation with the lowest binding free energy (kcal/mol) was selected for further analysis. The best-ranked ligand–receptor complexes were analyzed and visualized using Discovery Studio Visualizer [71], and two-dimensional interaction maps were generated to identify key molecular interactions.

### 4.9. Statistical Analysis

The concentration–mortality data from the *Ae. aegypti* adulticidal bioassays were subjected to Probit analysis using the PROBIT procedure in SAS (version 9.2, SAS Institute, Cary, NC, USA) to estimate lethal concentrations (e.g., LC_10_, LC_50_, and LC_80_) and their respective 95% confidence intervals. For the L4 larval susceptibility and *Ap. mellifera* mortality assays, data were first checked for normality (Shapiro–Wilk test) and homoscedasticity (Levene’s test). When assumptions were met, data were analyzed using one-way ANOVA, followed by Tukey’s HSD post hoc test (*p* < 0.05). Repeated measures ANOVA was employed for mortality and diet consumption in the selectivity bioassays of forager bees. For morphometric data, Procrustes ANOVA was performed using MorphoJ (version 1.10.08) to evaluate the significance of shape and size variation, as well as fluctuating asymmetry, considering individuals and sides as main effects. All biological replicates were treated as independent observations. General statistical analyses were performed using SigmaPlot (version 14.0) and SAS.

## 5. Conclusions

In conclusion, our study highlights that *S. guianensis* and *S. gesnerioides* essential oils exhibit adulticidal activity against *Ae. aegypti*. While *S. guianensis* essential oils exhibited remarkably higher adulticidal toxicity, early-life (i.e., from eggs to L2 larval phases) exposure to sublethal concentrations of *S. gesnerioides* essential oils was associated with reduced susceptibility at the L4 larval phase. This observed response is potentially linked to energetic reallocation that may be linked to wing deformations observed in emerged adults. Although our findings phenotypically support such a hypothesis, i.e., observed developmental instability and wing asymmetry, further mechanistic and multi-generational investigations are still required. Furthermore, in silico docking predictions indicated a potential interaction with mosquito TRPV channels, while initial laboratory screenings suggested low acute oral toxicity toward the non-target pollinator *Ap. mellifera*. Nevertheless, electrophysiological studies and broader ecological testing remain necessary to validate these potential mechanisms and safety profiles. Our findings provide evidence for the strategic potential of *Siparuna* essential oils as candidates for integrated vector management, impacting mosquito fitness beyond acute mortality.

## Figures and Tables

**Table 1:** Chemical composition, concentrations (%), and terpene classification for the essential oil of *Siparuna guianensis* and *Siparuna gesnerioides*.

N°	Compound	RI_cal_	RI_lit_	Class of Compound	*Siparuna guianensis*	*Siparuna gesnerioides*
					A_rel_ (%)	A_rel_ (%)
1	*α*-Pinene	916	932	MH	-	1.11
2	*α*-Thujene	924	924	MH	5.67	-
3	*β*-Pinene	957	974	MH	-	1.81
4	Limonene	1011	1024	MH	-	0.58
5	(*Z*)-Tagetone	1128	1148	MO	-	4.22
6	(*E*)-Tagetone	1136	1139	MO	-	6.16
7	Verbenone	1212	1204	MO	-	0.81
8	Undecan-2-one	1274	1293	K	-	1.11
9	U	1333	-	-	2.70	-
10	α-Ylangene	1350	1373	SH	-	0.86
11	α-Copaene	1359	1374	SH	8.33	5.66
12	*β*-Bourbonene	1368	1387	SH	6.21	0.74
13	(*E*)-Caryophyllene	1401	1417	SH	4.92	4.24
14	*α*-Bergamotene	1414	1411	SH	-	7.46
15	α-Himachalene	1426	1449	SH	-	1.41
16	α-Humulene	1431	1452	SH	-	2.85
17	U	1457	-	-	-	5.38
18	Germacrene D	1463	1484	SH	25.10	19.90
19	*β*-Selinene	1465	1489	SH	-	1.37
20	*α*-Acoradiene	1473	1464	SH	-	5.24
21	*α*-Amorphene	1478	1483	SH	-	2.06
22	Bicyclogermacrene	1479	1500	SH	42.93	-
23	*β*-Bisabolene	1487	1505	SH	-	2.80
24	*γ*-Muurolene	1491	1478	SH	-	3.95
25	*δ*-Cadinene	1501	1522	SH	-	7.92
26	U	1505	-	-	4.14	-
27	Valencene	1512	1496	SH	-	1.65
28	Selina-3,7(11)-diene	1519	1545	SH	-	3.38
29	Elemol	1526	1548	SO	-	2.02
30	Germacrene B	1533	1559	SH	-	1.87
31	Caryophyllene oxide	1558	1582	SO	-	0.68
32	*α*-Muurolol	1622	1644	SO	-	0.97
33	*β*-Eudesmol	1625	1649	SO	-	0.31
34	Guaiol	1628	1600	SO	-	1.48
Classes of compounds						
Monoterpene hydrocarbons (MH)					5.67	3.50
Oxygenated monoterpenes (MO)					-	11.19
Ketone (K)					-	1.11
Sesquiterpene hydrocarbons (SH)					87.49	70.06
Oxygenated sesquiterpenes (SO)					-	7.33
Total identified					93.16	94.62

U = unidentified; RI_cal_ = calculated retention index; and RI_lit_ = literature retention index [34].

**Table 2:** Fluctuating asymmetry (shape) by population. Results of Procrustes ANOVA performed independently for each experimental group (Control, DMSO, *Siparuna guianensis* essential oil; *Siparuna gesnerioides* essential oil). SS (Ind × Side) corresponds to the fluctuating asymmetry component; SS Total represents the total sum of squares for shape; FA Variation (%) indicates the percentage of variation attributable to fluctuating asymmetry; *F* (shape) corresponds to the F statistic of the Individual × Side term. All effects were statistically significant (*p* < 0.0001).

Groups	SS (Ind × Side)	SS Total	FA Variation (%)	*F* (Shape)	*p*-Value
control	0.0138	0.0667	20.68%	73.97	<0.0001
DMSO	0.0140	0.0661	21.15%	105.27	<0.0001
*Siparuna guianensis* essential oil	0.0165	0.0637	25.84%	87.82	<0.0001
*Siparuna gesnerioides* essential oil	0.0206	0.0811	25.35%	71.43	<0.0001

**Table 3:** Mahalanobis distances and Procrustes distances between populations. Mahalanobis distances are derived from canonical variation analysis (CVA) and reflect multivariate differentiation in shape space. Procrustes distances represent absolute divergence in configuration after generalized Procrustes superimposition.

Compaction	Distance of Mahalanobis	Distance of Procrustes
Control–DMSO	1.9363	0.0091
Control–*Siparuna guianensis* essential oil	1.9683	0.0073
Control–*S. gesnerioides* essential oil	2.1766	0.0102
DMSO–*S. guianensis* essential oil	1.8480	0.0089
DMSO–*S. gesnerioides* essential oil	1.5089	0.0079
*Siparuna guianensis* essential oil–*S. gesnerioides* essential oil	1.6396	0.0072

**Table 4:** Repeated measures analysis of variance for mortality and dietary intake of *Apis mellifera* exposed to essential oil of *Siparuna guianensis* (24.0 nL/mL) and *Siparuna gesnerioides* (364.0 nL/mL).

Sources of Variation	Diet Consumption				Mortality		
	df	*F*		*P*	*F*	*P*	
*Between samples*							
Essential oils (ET)	2	0.43		0.66	2.28	0.16	
Error	9	-		-	-	-	
	df_den_/df_num_	Wilks’ lambda	*F*_approx_	*P*	Wilks’ lambda	*F*_approx_	*P*
*Within Samples*							
Time (T)	9/1	0.8531	1.55	0.25	0.9861	0.13	0.73
ET x T	9/2	0.7541	1.47	0.28	0.7034	1.90	0.20

## Data Availability

The original contributions presented in this study are included in the article. Further inquiries can be directed to the corresponding authors.
